# Identification of novel *PANDAR* protein interaction partners involved in splicing regulation

**DOI:** 10.1038/s41598-018-21105-6

**Published:** 2018-02-12

**Authors:** N. Pospiech, H. Cibis, L. Dietrich, F. Müller, T. Bange, S. Hennig

**Affiliations:** 1 Chemical Genomics Centre of the Max-Planck Society, Otto-Hahn-Str. 11, 44227 Dortmund, Germany; 2 Max-Planck Institute of Molecular Physiology, Otto-Hahn-Str. 11, 44227 Dortmund, Germany; 3 Institute of Medical Psychology, LMU Munich, Goethe-Str. 31, 80336 Munich, Germany; 4 Vrije Universiteit Amsterdam, de Boelelaan 1108, 1081HZ Amsterdam, Netherlands

## Abstract

Interactions of long non-coding RNAs (lncRNA) with proteins play important roles in the regulation of many cellular processes. *PANDAR* (**P**romotor of *CDKN1A* **An**tisense **D**NA damage **A**ctivated **R**NA) is a lncRNA that is transcribed in a p53-dependent manner from the *CDKN1A* promoter and is involved in the regulation of proliferation and senescence. Overexpression of *PANDAR* has been observed in several tumor species and correlated with a poor prognosis for patient survival rate. Depending on the cellular state, *PANDAR* is known to interact with proteins such as the nuclear transcription factor Y subunit A (NF-YA) and the scaffold attachment factor A (SAF-A). However, a comprehensive analysis of the *PANDAR* interactome was missing so far. Therefore, we applied peptide nucleic acid (PNA)-based pull-downs combined with quantitative mass spectrometry to identify new protein binding partners. We confirmed potential candidates like U2AF65 and PTBP1, known to be involved in RNA processing. Furthermore, we observed that overexpression of *PANDAR* leads to a reduced level of the short pro-apoptotic *BCL-X* splice variant (*BCL-XS*) which is regulated by PTBP1. Simultaneous overexpression of PTBP1 was able to rescue this effect. Overall, our data suggest a role for *PANDAR* in the regulation of splicing events via its interaction partner PTBP1.

## Introduction

Non-coding RNAs (ncRNAs) regulate different cellular mechanisms by interacting with all kind of biomolecules, such as other nucleic acids, proteins or small molecules and co-factors, thereby modifying their subsequent functions^1–3^. Those ncRNAs have a low potential to be translated into proteins and can be sub-divided according to their size into small (<200 nt) and long non-coding RNAs (>200–100,000 nt, lncRNAs). The function and cellular role of lncRNAs is diverse due to the various different interaction mechanisms and partners^3–5^. Their expression profile can vary within different cell types, developmental states and diseases^6,7^. Therefore, the distinct expression profile of lncRNAs is often used as biomarker for disease type and stage. Considering the plethora of identified lncRNAs, there are only a few well-characterized species, yet. This makes the functional analysis including the involved complexes and binding partners an essential task for research^8–11^.

The lncRNA *PANDAR* (**P**romotor of *CDKN1A* **An**tisense **D**NA damage **A**ctivated **R**NA) comprises 1506 nt and is transcribed in antisense direction from the *CDKN1A* promotor. When bound to protein complexes, *PANDAR* is able to modulate different cellular effects: the interaction with the NF-YA protein, for instance, leads to the inhibition of apoptotic gene activation^12^. Also, the scaffold attachment factor A (SAF-A) interacts with *PANDAR* and plays an important role within the regulation of cellular senescence^13^. In proliferative fibroblast cells, *PANDAR* and SAF-A recruit the polycomb repressive complexes PRC1 and 2 to inhibit the transcription of pro-senescent genes. Contrary to this, in senescent fibroblast cells, *PANDAR* interacts with NF-YA thereby sequestering it from promotor regions of proliferation responsible genes^13,14^.

The investigation of patient derived non-small cell lung carcinoma samples revealed decreased *PANDAR* levels compared to adjacent healthy tissue. This correlates with increased tumor size and tumor-nodus-metastasis (TNM) stage^15^. In contrast to this, several cancer types like gastric cancer, hepatocellular carcinoma and bladder cancer revealed increased *PANDAR* levels that correlated with a higher TNM stage and poor prognosis^16–18^. Further *in vitro* analysis of bladder cancer revealed that *PANDAR* upregulation leads to proliferation, while *PANDAR* knockdown results in apoptosis^18^. Given the variety of tightly regulated expression levels and involvements within various cellular stages, the detailed interaction network of *PANDAR* and its mechanisms behind needs further investigations.

Herein, we identified *PANDAR* interacting proteins using a peptide nucleic acid (PNA)-based pull-down combined with quantitative mass spectrometry. Subsequent functional characterization of interacting candidates revealed a potential role of *PANDAR* in pre-mRNA splicing regulation via one of its newly identified binding partners polypyrimidine tract-binding protein 1 (PTBP1).

## Results

### Identification of the lncRNA *PANDAR* interactome from human osteosarcoma cells

**Figure 1:**
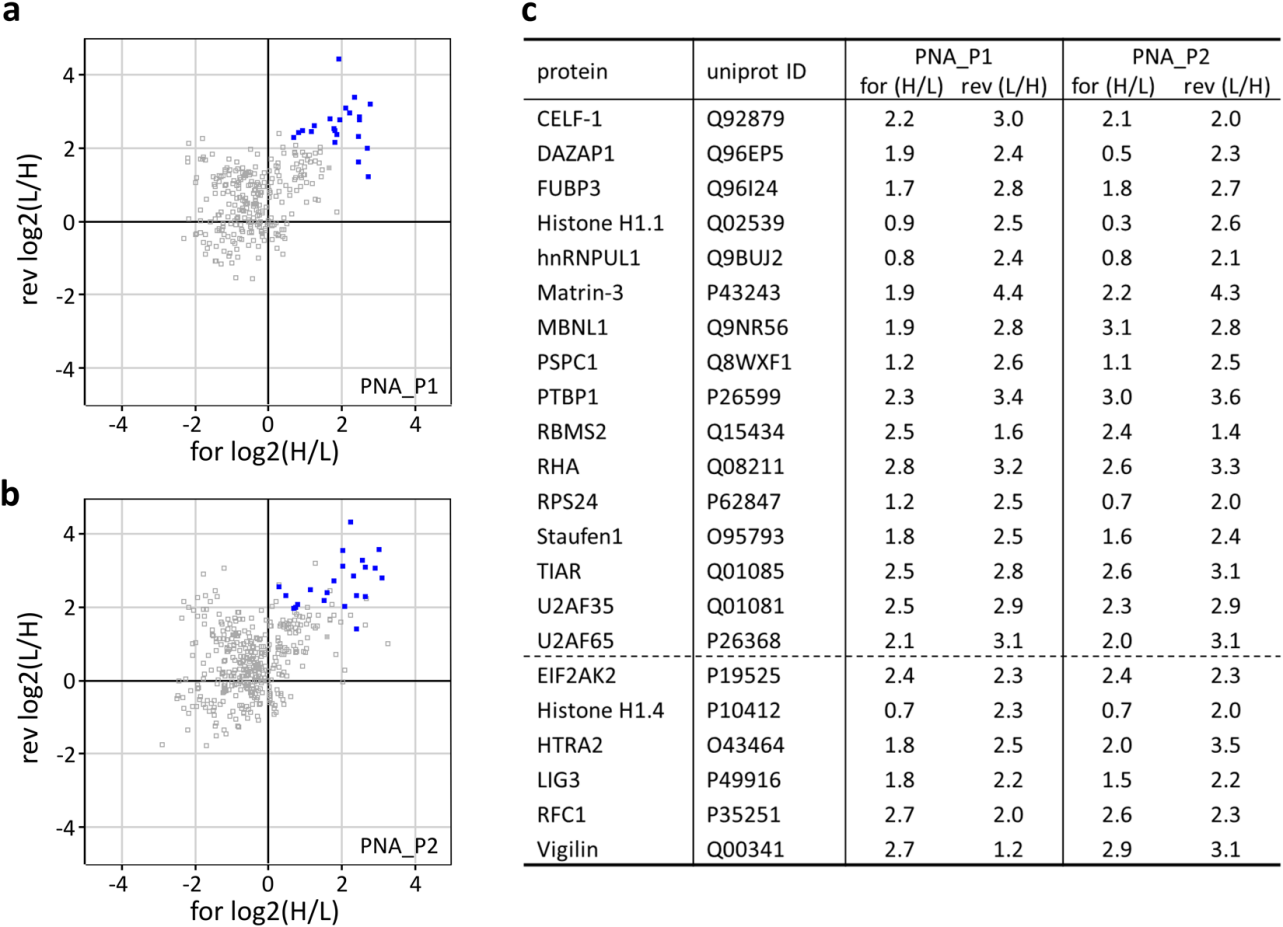
Potential *PANDAR* binding partners by PNA-based pull-down with quantitative mass spectrometry. *PANDAR* targeting PNA-pull-down experiments from SILAC treated U2 OS cells were performed utilizing biotinylated PNA_P1 (**a**) or PNA_P2 (**b**) probe. Log2 heavy to light ratios from quantitative mass spectrometric analysis of the forward (H/L) and reverse (L/H) experiments are plotted against each other. Potential *PANDAR* binding candidates (depicted in blue) appear in the upper right quadrant of the plot. Selected candidates for interaction were required to be quantified in both probes PNA_P1 and PNA_P2 with at least one log2 value >2 (4-fold binding difference). (**c**) Resulting hit candidates are listed. Candidates below the dashed line were excluded from further experiments due to technical reasons.

To specifically identify *PANDAR* bound proteins, we used the combination of a peptide nucleic acid (PNA)-based *PANDAR* pull-down followed by quantitative mass spectrometry after stable isotope labeling in cell culture (SILAC). Therefore, we designed, synthesized and analyzed *PANDAR* specific PNA probes (Supplementary Fig. 1a). These were able to enrich *PANDAR* from total isolated RNA samples (Supplementary Fig. 1b) and showed affinities with the best (PNA_P1) reaching down to the lower nM range (1.8 nM, Supplementary Fig. 1c). To identify potential interaction partners, lysates from osteosarcoma cells grown in either “heavy” media or “light” media (forward experiment) were supplemented with or without PNA-probe for affinity pull-down via magnetic beads. The sample without PNA-probe served as negative control. Additionally, the same experiment was conducted with exchanged settings (reverse experiment) (Supplementary Fig. 2). Pull-downs were subsequently subjected to quantitative mass spectrometry (Fig. 1). For proteins to be selected as possible binding partners, we claimed that the difference between control and probe was at least four fold (log2 heavy to light (H/L) ratio > 2) in either the forward or reverse experiment (log2 (L/H) > 2). In addition, we required the candidates to be identified and quantified with both probes in independent experiments (Fig. 1a,b). Taken together, we determined 22 potential binding candidates for *PANDAR* (Fig. 1c).

### Validation of novel *PANDAR* interaction partners

**Figure 2:**
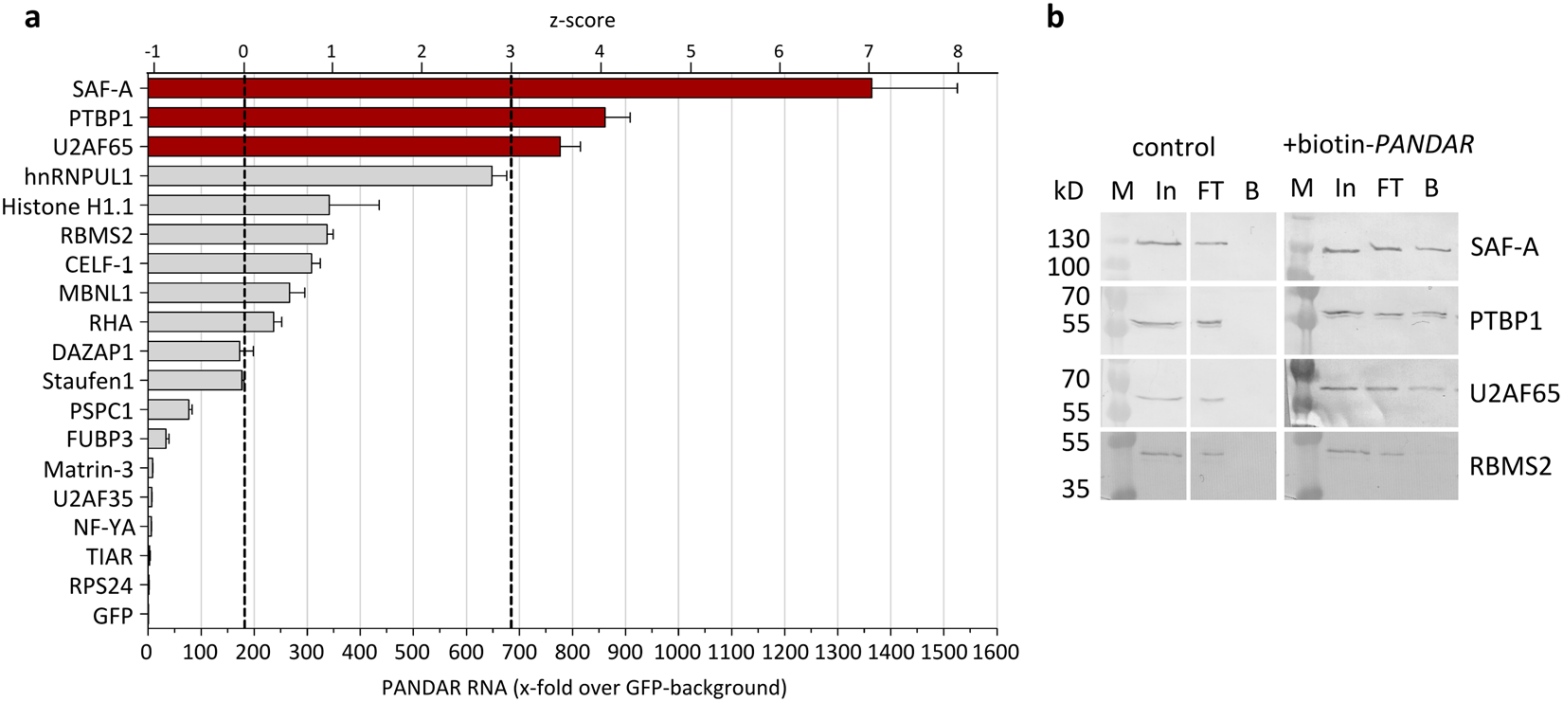
Validation of *PANDAR*-protein complexes. (**a**) eGFP fused protein candidates were analyzed with respect to their *PANDAR* binding ability by RT-qPCR. Bound *PANDAR* RNA was determined as fold-enrichment compared to eGFP negative control (=1). Three positive *PANDAR* binding proteins candidates (dark red bars) exceed the MAD of the population by a factor of >3 (z-score > 3), median = 177, MAD = 168. (**b**) Analysis of bound proteins to biotinylated *PANDAR* RNA. Western blot analysis for each of the binding candidates revealed binding of SAF-A, PTBP1 and U2AF65, but not for RBMS2 (z-score = 1) as internal negative control. In: input, FT: flowthrough, B: bound fractions.

To further validate potential *PANDAR* interaction partners, stably *PANDAR* overexpressing U-2 OS cells were transiently transfected with plasmids of eGFP-fusion proteins and subjected to GFP-RNA immunoprecipitation (GFP-RIP, Supplementary Fig. 3). Quantification of bound *PANDAR* was determined by reverse transcription-quantitative PCR (RT-qPCR) as relative enrichments over unspecific background of the eGFP-negative control. Additionally, we included NF-YA and SAF-A for the analysis, as these proteins were known to interact with *PANDAR*^12,13^. A threshold for most promising interaction partners was set to exceed the median absolute deviation (MAD) from the median of all candidates by a factor of three (z-score ≥ 3). SAF-A, PTBP1 and splicing factor U2AF 65 kDa subunit (U2AF65) fulfilled these criteria so that our subsequent analyses focused on these candidates (Fig. 2a).

For further cross-validation, we wanted to know if endogenous protein expression levels are sufficient to form the RNA-protein complex. Therefore, biotinylated *PANDAR* was bound to streptavidin magnetic beads and incubated with cell lysates derived from U-2 OS cells. Subsequent western blotting using protein-specific antibodies showed no binding for the negative control RNA-binding motif, single-stranded-interacting protein 2 (RBMS2, z-score = 1.0, Fig. 2b). In contrast, binding of the already known *PANDAR* interaction partner SAF-A was verified. Importantly, we confirmed PTBP1 and U2AF65 as two novel interaction partners of *PANDAR*.

### *PANDAR* plays a regulatory role in splicing by modulating PTBP1

**Figure 3:**
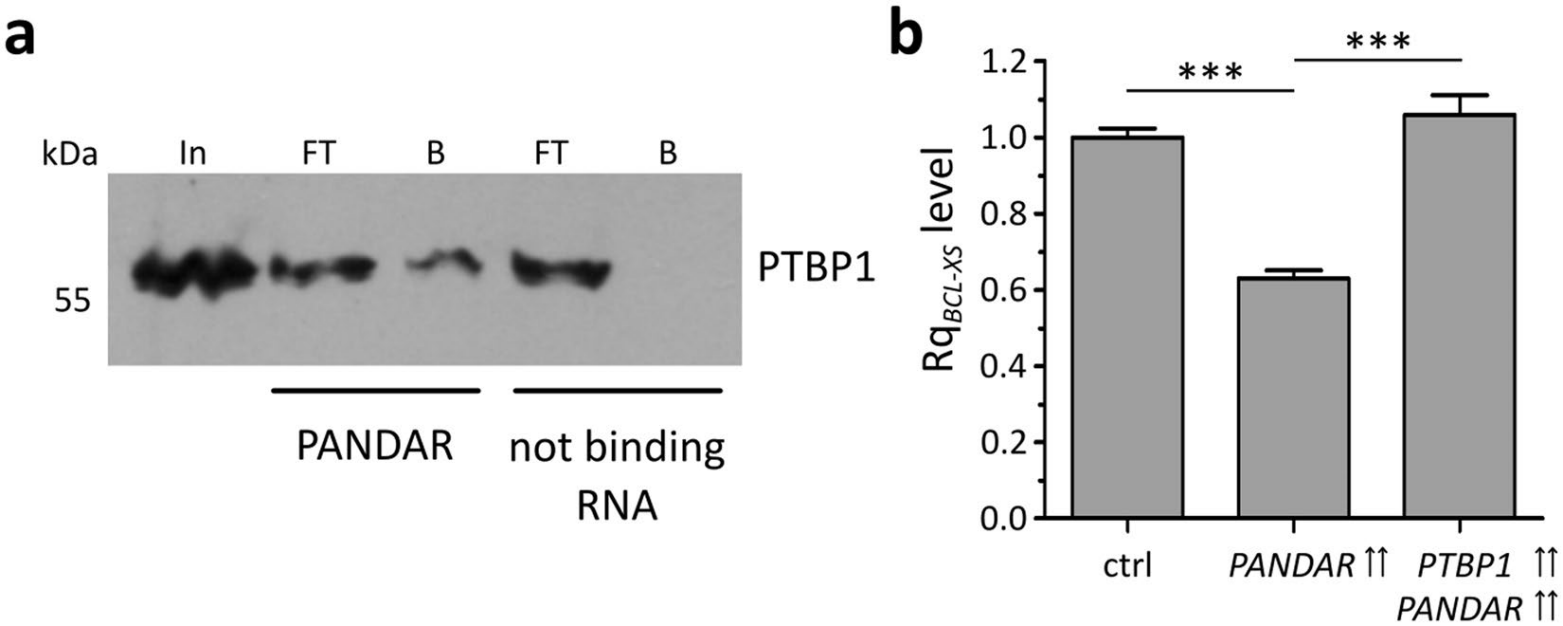
*PANDAR* induced *BCL-XS* mRNA reduction rescued by PTBP1. (**a**) PTBP1 western blot analysis of biotin-*PANDAR* pull-down samples from U-2 OS cell lysates revealed an enrichment of PTBP1 for *PANDAR* full length RNA. No PTBP1 enrichment was observed for the pull-down with non binding RNA control. (**b**) RT-qPCR analysis of *BCL-XS* mRNA level upon *PANDAR* overexpression in comparison to simultaneous overexpression of PTBP1 in U-2 OS cells. Shown are mean ± s. d.; n = 3; *p < 0.05, **p < 0.01, ***p < 0.001, n. s. = not significant; e. v. = empty vector control; Rq = relative quantity.

For functional analysis we next focused on PTBP1 as the candidate that showed the strongest signal in our SILAC experiment (Fig. 1) as well as the highest *PANDAR* binding capacity (Fig. 2). Previously, PTBP1 has been shown to be involved in the post-transcriptional splicing process of the *BCL-X* pre-mRNA towards the pro-apoptotic BCL-XS variant^19^. To further investigate if *PANDAR* is involved in the regulation of this process, we focused on the *PANDAR*-PTBP1 interaction. First, we performed *PANDAR*-PTBP1 interaction pull-down experiments with biotinylated full length *PANDAR* from U-2 OS cell lysate (Fig. 3a). Western blot analysis confirmed the interaction of the splicing regulator PTBP1 with *PANDAR*, but not with a non-binding control RNA. As PTBP1 regulates *BCL-X* alternative splicing, we asked if the interaction with *PANDAR* is able to modulate this alternative splicing of the *BCL-XS* isoform. We transiently overexpressed *PANDAR* in U-2 OS cells and analyzed the *BCL-XS* mRNA level quantitatively. We found the *BCL-XS* mRNA level to be significantly reduced (about 40%) compared to an empty vector control (Fig. 3b). Importantly, simultaneous PTBP1 overexpression fully rescued this *PANDAR* induced reduction of *BCL-XS* mRNA (Fig. 3b).

### Multiple PTBP1 binding regions of *PANDAR* are needed for *BCL-XS* mRNA reduction in U-2 OS cells

**Figure 4:**
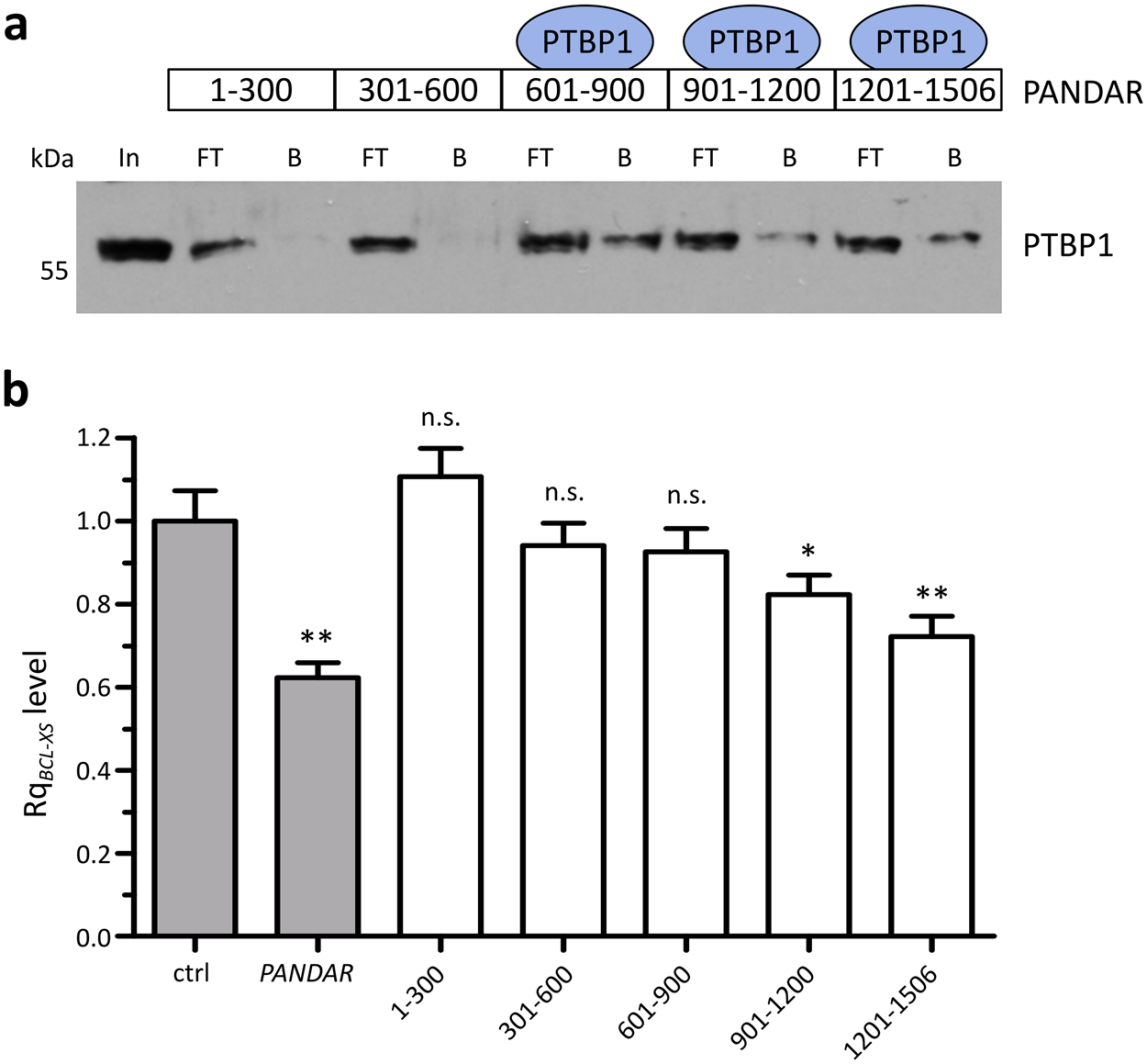
PTBP1 binds to multiple *PANDAR* regions. (**a**) PTBP1 western blot analysis of pull-down with different biotinylated *PANDAR* fragments revealed an enrichment of PTBP1 for *PANDAR* fragments 600–900 nt, 901–1200 nt and 1201–1506 nt, as depicted in the schemeatic overview above. In: input, FT: flowthrough, B: bound fraction. (**b**) *BCL-XS* mRNA level are analyzed by RT-qPCR upon overexpression of *PANDAR* full length in comparison to *PANDAR* fragments of 300 nt. Shown are mean ± s. d.; n = 3; *p < 0.05, **p < 0.01, ***p < 0.001, n. s. = not significant; e. v. = empty vector control; Rq = relative quantity.

For a more detailed mapping of *PANDAR* regions involved in PTBP1 binding, biotinylated *PANDAR* fragments each with a size of 300 nt were used for subsequent analysis (Fig. 4a). The respective *PANDAR* fragments were bound to streptavidin magnetic beads and used to analyze their ability to interact and therefore enrich PTBP1 from U-2 OS cell lysate. Western blot analysis revealed no PTBP1 interaction with the 5′ *PANDAR* regions up to 600 nt, while the 3′ fragments 601–900 nt, 901–1200 nt and 1201–1506 nt were able to enrich PTBP1 (Fig. 4a). To address, which of the PTBP1 binding regions of *PANDAR* are needed for the reduction of *BCL-XS* level, we overexpressed each of these fragments in U-2 OS cells and quantified the *BCL-XS* mRNA content (Fig. 4b). Here we could reproduce our previously observed 40% reduction of the *BCL-XS* mRNA level upon *PANDAR* overexpression. The *PANDAR* fragment analysis showed, that most of the *PANDAR* fragments show a slightly reduce *BCL-XS* mRNA levels (Fig. 4b). Notably, the most 3′-fragment (1200–1506 nt) shows the biggest effect of all tested fragments (about 30%). Still, more than one of the tested fragments seems to be needed to reach the highest *BCL-XS* reduction under these experimental conditions.

## Discussion

By use of the PNA-based *PANDAR* pull-down followed by quantitative mass spectrometry after SILAC, we were able to determine 22 potential binding candidates of the *PANDAR* interactome. Depending on the cellular state, the *PANDAR* expression pattern varies significantly. In proliferative fibroblast, for instance, *PANDAR* prefers to interact with SAF-A, while in senescent fibroblasts *PANDAR* interacts with NF-YA^12,13^. This is supported by our data which verify SAF-A as a *PANDAR* interactor, whereas NF-YA could not be found in this complex within these cells (Figs 1 and 2). On the basis of further interaction analysis, we could identify PTBP1 and U2AF65 as additional *PANDAR* interaction partners. Regarding all other candidates detected in our quantitative mass spectrometry analysis, an interaction with *PANDAR* is still possible. Their low *PANDAR* binding capacity in our GFP-RIP experiment could be due to a low affinity or an indirect binding via other mediator complexes.

U2AF65 has been shown to interact with the lncRNAs *MALAT1* and *NEAT1*. Thereby, it is retained in sub-nuclear structures and prevented from the recruitment to pre-mRNA^20,21^. In general, the U2AF65 subunit recognizes polypyrimidine tracts near 3′-splice sites and plays an important role in the assembly of the spliceosome^22–24^. Since those polypyrimidine tracts are also targeted by PTBP1, it has been suggested, that PTBP1 competes with U2AF65 for its binding and regulates alternative splicing processes in this way^25,26^. Notably, the detailed orchestration of U2AF65 together with PTBP1 in context with *PANDAR* and its role in splicing regulation needs thorough investigations in the future.

As PTBP1 is our strongest validated candidate (Figs 1 and 2), we focused on its functional role upon *PANDAR* binding. PTBP1 has been shown to bind to a lncRNA *Pnky* and regulate expression and splicing of transcripts involved in neuronal differentiation in mouse studies^27,28^. A comparison between *PANDAR* and *Pnky* does not show any identical regions on sequence level still they might be involved in a similar cellular process. As a member of the hnRNP family, PTBP1 is known to be involved in alternative splicing. PTBP1 has been shown to be involved in the regulation of post-transcriptional alternative splicing of the *BCL-X* pre-mRNA^19^. Depending on the proteins interacting with this pre-mRNA, *BCL-X* is spliced into a short (*BCL-XS*) or large (*BCL-XL*) variant. While the interaction of Serine/arginine splicing factor 1 leads to anti-apoptotic BCL-XL, its displacement by PTBP1 leads to the pro-apoptotic BCL-XS variant^19^. Based on this, we investigated if *PANDAR* interaction modulates the alternative splicing of *BCL-X* which is regulated by PTBP1. We could show that overexpression of *PANDAR* leads to a reduced *BCL-XS* mRNA. This could on the one hand be due to a decoy mechanism of *PANDAR*, on the other hand the function of other binding partners might be altered, which could cause this effect. However, PTBP1 alone rescues the effect and leads to a normal *BCL-XS* mRNA level, which strengthens the role of PTBP1 in this PANDAR induced splicing regulation.

Our mapping studies revealed a preferential interaction of PTBP1 to *PANDAR*s 3′-end (Fig. 4a). Here, several pyrimidine-rich elements are located, which could resemble the binding regions for PTBP1 binding with its RNA recognition motifs^29^. It is well known, that in most cases a sequence specific recognition with a high affinity is not possible for a single RNA binding domain^30^. Therefore, multiple domains are often required to recognize a longer sequence of RNA. This goes in line with our observation, that multiple PTBP1 binding regions of *PANDAR* are needed to observe the *BCL-XS* mRNA splicing effect to the full extend (Fig. 4b).

**Figure 5:**
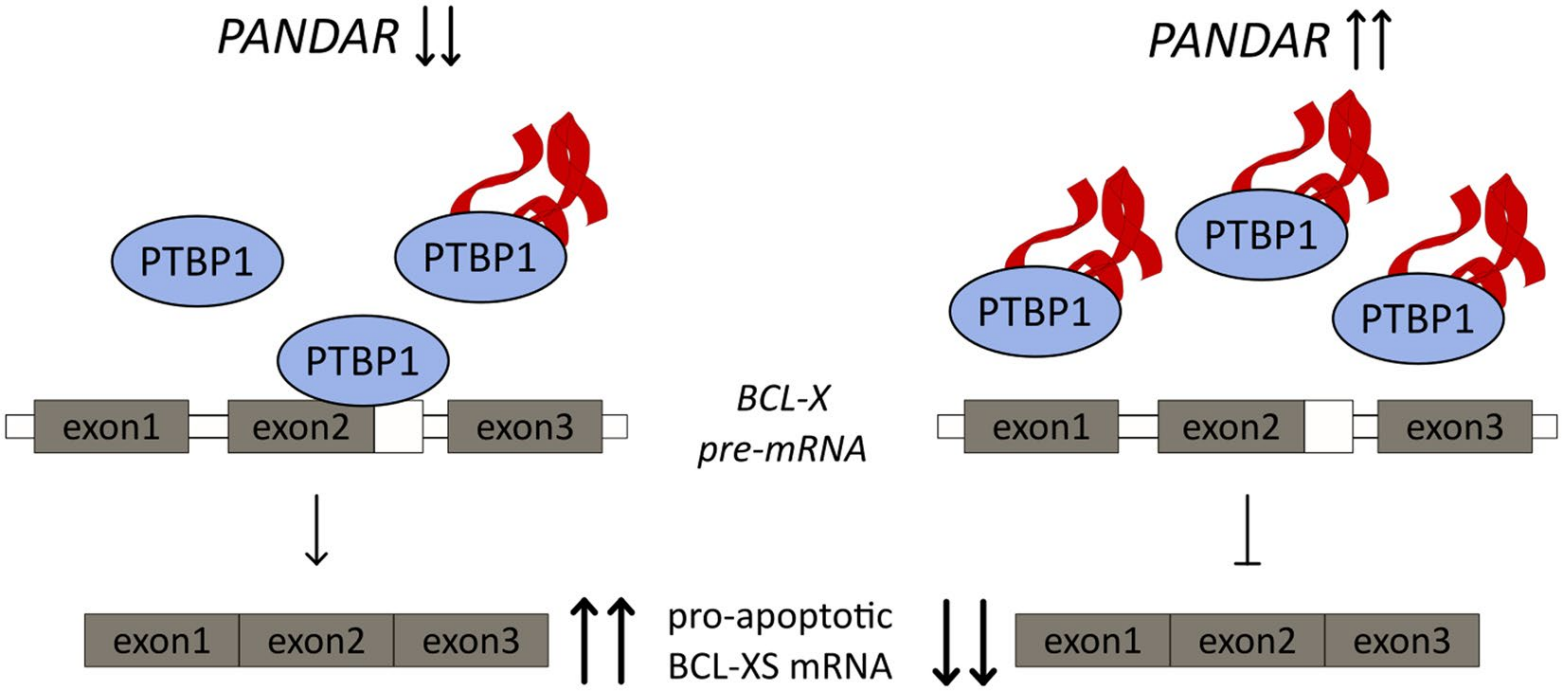
Model of *PANDAR* involved in splicing regulation. At normal and low *PANDAR* level, PTBP1 is involved in splicing of the *BCL-XS* short isoform resulting in its upregulation and pro-apoptotic cellular outcome (left). Upon *PANDAR* upregulation, *PANDAR* is bound to PTBP1 and functions as a decoy resulting in a decreased level of *BCL-XS* splice variant leading to a reduced level of pro-apoptotic response (right). Red ribbon: schematic representation of *PANDAR*.

On the basis of these findings, we propose that the lncRNA *PANDAR* is involved in the regulation of alternative splicing of *BCL-XS* mRNA. In a cell with a regular *PANDAR* RNA expression, PTBP1 is bound to *BCL-X* pre-mRNA to regulate the alternative splicing of pro-apoptotic *BCL-XS* mRNA (Fig. 5, left). In contrast to this, in a cell with upregulated *PANDAR* levels, *PANDAR* might act as a decoy for PTBP1 and thereby reducing the *BCL-XS* mRNA variant (Fig. 5, right). The latter could be an explanation for the higher TNM-stages and poor prognosis of *PANDAR* upregulated cancer types^16–18^. In this scenario, *PANDAR* could play an important key role by reducing the apoptotic response, based on the decreased pro-apoptotic BCL-XS level.

It becomes a compelling biological question for the future to investigate if *PANDAR* has a broader role and might be a general regulator of alternative splicing by interacting with various splicing proteins. In addition, the modulation of each interaction partner by *PANDAR* needs further functional analysis, focusing on how it might affect binding partners, localization and cellular behavior. Our new identified *PANDAR* interactions might open up a new important layer of regulation for the binding partners and also *PANDAR* itself.

Since *PANDAR* is known to be upregulated in several cancer types, it would be of utmost importance to investigate, if the identified RNA-protein interactions contribute to the pathological phenotype and might even open up the possibility for new therapeutic targets.

## Methods

### PNA synthesis and affinity determination via fluorescence polarization assay

Peptide nucleic acids (PNA) were specifically designed against the *PANDAR* sequence (NR_109836.1). A secondary structure model of PANDAR (mfold, Supplementary Fig. 1a) was used to design binding PNA probes hybridizing to open loop conformations that are found in the core body of the RNA (P1: *PANDAR* 1437–1451 and P2: *PANDAR* 458–472). A size of 15 bases each was chosen to give enough affinity for the target RNA: PNA_P1 (C-TTGGAGCTGGAGTCA-N) and PNA_P2 (C-CGTCTGTGTTACCGA-N). The synthesis was performed by solid phase synthesis with a NovaSynTGR resin with PNA building blocks (Fmoc-G(Bhoc)-aeg-OH, Fmoc-C(Bhoc)-aeg-OH, Fmoc-A(Bhoc)-aeg-OH, Fmoc-T-aeg-OH) purchased from Link Technologies. Each PNA was coupled to a PEG5 linker (Iris Biotech) and depending on the purpose of use, additionally to either biotin or FITC (Sigma Aldrich). PNA was purified and binding affinity of PNA to *PANDAR* RNA was analyzed by fluorescence polarization (FP) (Tecan Safire2, λ_Ex_: 470 nm, λ_Em_: 525 nm). Therefore, *in vitro* transcribed full length *PANDAR* RNA (1506 nt) was serial diluted against 10 nM of FITC-PNA_P1 or FITC-PNA_P2 in 10 mM HEPES pH 7.5, 150 mM NaCl, 0.5 mM EDTA, 0.01% Tween20. After 3 h of incubation on ice the FP was measured and K_D_ was analyzed.

### Cell culture

Human osteosarcoma cells (U-2 OS) were cultured in Dulbecco’s modified Eagle’s medium (DMEM, PAN Biotech) supplemented with 10% fetal bovine serum (PAN Biotech) and incubated in a humidified chamber with 5% CO_2_ at 37 °C.

### PNA pull-down from total isolated RNA

To analyze the binding of PNA to RNA from total isolated RNA, pull-down experiments were performed. First 50 pmol biotinylated PNA and 5 µg total isolated RNA from stable *PANDAR* overexpressing U-2 OS cells were incubated for 3 h at 4 °C on a rotating wheel in 10 mM HEPES pH 7.5, 150 mM NaCl, 0.5 mM EDTA. Next, PNA-RNA complexes were added to Streptavidin magnetic beads (NEB) and incubate for 1 h at 4 °C on a rotating wheel. After magnetic separation and washing, the bound RNA was reverse transcribed into cDNA (Quanti Tect Reverse Transcription Kit, Qiagen) and subsequently used for quantitative PCR (RT-qPCR) (SensiMix SYBR Low-ROX Kit, Bioline) in an Applied Biosystems 7500 Fast Real-Time PCR System (Thermo Fisher Scientific). A *PANDAR* coding plasmid was used as a standard.

### Peptide nucleic acid-stable isotope labeling by amino acids in cell culture (PNA-SILAC)

To determine *PANDAR* binding candidates within osteosarcoma cells, cells were cultivated in specialized SILAC medium (DMEM, E15-086, PAA; dialyzed serum, A11-107, PAA) without arginine and lysine supplemented with either “light” arginine and lysine (referred to as Arg0/Lys0; 42 mg/l Arg0 and 73 mg/l Lys0 were used; A6969, L8662, Sigma Aldrich) or “heavy” arginine and lysine (referred to as Arg10/Lys8; 42 mg/l ^13^C_6_^15^N_4_, Arg10 and 73 mg/l ^13^C_6_^15^N_2_ Lsy8 were used, Silantes) for 5 passages as described before^31^. After incorporation of amino acids into the proteins, cell lysates were spiked with *in vitro* transcribed *PANDAR* full length RNA and pulled down with biotinylated PNA pull-down probes (PNA_P1 or PNA_P2). In detail, 50 pmol biotinylated PNA was bound to Streptavidin magnetic beads for 1 h at 4 °C on a rotating wheel. The unbound PNA in the supernatant was removed and 100 pmol *in vitro* transcribed *PANDAR* full length RNA was incubated for 1.5 h at 4 °C. The control contained only magnetic beads. Cell pellets were lysed with lysis buffer (50 mM Tris-Cl pH 8.0, 150 mM NaCl, 0.1% (w/v) SDS, 1% (v/v) NP40, 1 mM PMSF, 1x complete EDTA free protease inhibitor cocktail (Roche)) for 30 min on ice. To get rid of cell debris, the suspension was centrifuged at 15000 × g for 10 min at 4 °C. To get rid of unbound RNA, PNA-RNA complexes bound to the beads were washed with wash buffer (50 mM TrisCl pH 8.0, 150 mM NaCl, 1x complete EDTA free protease inhibitor cocktail (Roche)) and the soluble supernatant of the lysates was added to the respective samples. After incubation (1.5 h, 4 °C), samples were magnetically separated and washed three times. After the first wash, samples were combined for forward and reverse experiments respectively in new tubes (Supplementary Fig. 2): Arg0/Lys0 lysate without spiked *PANDAR* (beads only) and Arg10/Lys8 lysate with spiked *PANDAR* (referred to as “for” experiment) and in parallel Arg0/Lys0 lysate with spiked *PANDAR* and Arg10/Lys8 lysate without spiked *PANDAR* (beads only) (referred to as “rev” experiment). Afterwards, samples were subjected to quantitative mass spectrometry.

### Quantitative mass spectrometry

Samples were reduced, alkylated and digested directly on beads with LysC/Trypsin and prepared for LC-MS/MS analysis as previously described^32^. Digested samples were separated on a Thermo Fisher Scientific EASY-nLC 1000 HPLC system (Thermo Fisher Scientific, Odense, Denmark) using a two hours gradient from 5–60% acetonitrile with 0.1% formic acid and directly sprayed in a quadrupole Orbitrap mass spectrometer (Q Exactive, Thermo Fisher Scientific) via a nano-electrospray source^33^. The Q Exactive was operated in data-dependent mode acquiring one survey scan and ten subsequent MS/MS scans^34^. Resulting raw files were processed with the MaxQuant software (version 1.5.2.18) using the uniprot human database for the search giving deamidation (NQ), oxidation (M) and acetylation at the N-terminus as variable modifications and carbamidomethylation (C) as fixed modification^35^. A false discovery rate cut off of 1% was applied at the peptide and protein levels. For quantitation we filtered for at least two SILAC ratio counts per protein.

### GFP-RNA-immunoprecipitation (GFP-RIP)

pDest-eGFP-CUGBP1 and pDest-eGFP-MBNL1 were gifts from Nicolas Charlet-Berguerand (Addgene plasmid #61276 and #61277), pcDNA3-HtrA2-eGFP was a gift from L. Miguel Martins^36^. All other potential *PANDAR* binding candidates were sub-cloned from cDNA into a pcDNA3 mammalian expression vector (Thermo Fisher Scientific), which was modified with an N-terminal eGFP-coding sequence and to be compatible for gateway cloning (Thermo Fisher Scientific). Clones were sequenced and used for transient transfection with Lipofectamin2000 (Invitrogen) for 24 h in a stable *PANDAR* overexpressing U-2 OS cell line. For each experiment, a eGFP empty vector negative control (pcDNA3-eGFP) was used. After checking the transfection efficiency, cells were washed twice with ice cold PBS and harvested by scraping at 4 °C in lysis buffer (50 mM Tris-Cl pH 8.0, 150 mM NaCl, 0.1% (w/v) SDS, 1% (v/v) NP40, 1 mM PMSF, 1x complete EDTA free protease inhibitor cocktail (Roche)). Suspension was incubated on ice for 30 min and shred by using a sterile syringe (24G). Cell lysates were centrifuged for 10 min at 20000 rpm at 4 °C. The supernatant was added to equilibrated GFP-MA-Trap Beads (ChromoTek) and incubated for 2 h on a rotating wheel at 4 °C. After magnetic separation, the flowthrough was harvest to analyze the saturation of eGFP-protein binding to the beads. Afterwards, beads were washed three times with ice cold buffer (10 mM HEPES pH 7.5, 150 mM NaCl, 0.5 mM EDTA) and split for RNA and protein analysis. Binding of the eGFP-tagged proteins was confirmed by western blotting using an anti-GFP antibody (Table 1). Therefore, proteins were separated by SDS-PAGE and transferred to nitrocellulose membranes (GE Healthcare) via semidry method with the Trans-Blot SD Semi-Dry Transfer Cell (Bio-Rad). Next, membranes were incubated with 5% milk powder in TBS-T for 1 h at room temperature followed by the incubation with the primary antibody in 5% milk in TBS-T over night at 4 °C. After three washing steps with TBS-T, membranes were incubated with the corresponding alkaline phosphatase-conjugated secondary antibody in 5% milk in TBS-T for 1 h at room temperature. After three washing steps with TBS-T, western blots were developed by using BCIP/NBT solution (Merck).

**Table 1:** Antibodies.

name	company
rabbit anti-PTBP1 (12582-1-AP)	Proteintech
rabbit anti-U2AF65 (15624-1-AP)	Proteintech
rabbit anti-SAF-A (14599-1-AP)	Proteintech
rabbit anti-RBMS2 (13166-1-AP)	Proteintech
rabbit anti-GFP (AS-29779)	Eurogentech
goat anti rabbit AP-linked (sc-2007)	Santa Cruz Biotechnology
goat anti rabbit HRP-linked (AQ123P)	Millipore

RNA samples were purified by phenol-chloroform extraction. To analyze the bound *PANDAR* RNA, samples were subjected to RT-qPCR and absolute values determined via a *PANDAR* coding plasmid. Relative enrichments were calculated over eGFP background control. Promising interaction partner should exceed the median absolute deviation (MAD) from the median of the population by a factor of three (z-score ≥ 3).

### Biotinylated-*PANDAR* pull-down

The candidates with highest *PANDAR* enrichment were validated by pull-down from osteosarcoma cell lysate utilizing *in vitro* biotinylated *PANDAR* RNA. Therefore, *in vitro* transcription was performed with T7-transcription kit (Thermo Fisher Scientific) and biotin RNA labeling mix (Roche) for 2 h at 37 °C and DNase treated for 45 min at 37 °C. The RNA was purified with RNA Clean and Concentrator5 (ZymoResearch). The RNA was heated up to 50 °C for 5 min, chilled for 2 min on ice and 15 min at room temperature in 10 mM Tris-Cl pH 7.0, 100 mM KCl, 10 mM MgCl_2_. Equilibrated streptavidin magnetic beads (Dynabeads, Thermo Fisher Scientific) were incubated with the biotinylated *PANDAR* RNA for 1.5 h at 4 °C on a rotating wheel. To rule out unspecific binding of proteins to streptavidin magnetic beads, beads without RNA were used. The supernatants of cell lysates (as described before) were incubated with either *PANDAR* coated or empty beads for 2 h at 4 °C on a rotating wheel. After magnetic separation, beads were washed four times with ice cold buffer and resuspended in 1xSDS sample buffer for further western blot analysis (antibodies: Table 1). Western blotting was performed as described above, but membranes were incubated with a HRP-linked secondary antibody and developed via ECL method on an autoradiography film.

### Analysis of *BCL-XS* mRNA level

The *PANDAR* full length sequence was sub-cloned downstream of a CMV promoter into a modified pcDNA3 vector backbone which additionally carried a eYFP as transfection control (pcDNA3-TcYPF-*PANDAR*). All PANDAR fragments (containing the nucleotides 1–300, 301–600, 601–900, 901–1200 and 1201–1506) where sub-cloned via Gateway cloning (Thermo) into a Gateway compatible pcDNA3-GW vector backbone. Osteosarcoma cells (U-2 OS) were co-transfected with pcDNA3-TcYPF-*PANDAR* (or PANDAR fragment containing pcDNA3 plasmids) and either pcDNA3-eGFP-PTBP1 or an empty control plasmid (pcDNA3-eGFP) for 48 h using Lipofectamin 2000 (Invitrogen). Total RNA was extracted (Quick-RNA MicroPrep Kit, Zymo Research), reverse transcribed into cDNA (Quanti Tect Reverse Transcription Kit, Qiagen) and subsequently used for quantitative PCR (SensiMix SYBR Low-ROX Kit, Bioline) in an Applied Biosystems 7500 Fast Real-Time PCR System (Thermo Fisher Scientific). Utilized primer pairs are listed in Table 2. For relative quantification 2^−ΔΔC^_T_ method was used with *GAPDH* as reference gene^37^.

**Table 2:** qPCR primer.

name	sequence (5′ → 3′)
PANDAR_forward	TGCACACATTTAACCCGAAG
PANDAR_reverse	CCCCAAAGCTACATCTATGACA
BCL-XS_forward	GAGCTTTGAACAGGATACTTTTGTGG
BCL-XS_reverse	GAAGAGTGAGCCCAGCAGAAC
GAPDH_forward	TGCACCACCAACTGCTTAGC
GAPDH_reverse	GGCATGGACTGTGGTCATGAG

### Data availability

All data generated or analyzed during this study are included in this published article (and its Supplementary Information files).

## Electronic supplementary material

Supplementary Information

## References

[1] Prensner JR, Chinnaiyan AM (2011). The emergence of lncRNAs in cancer biology. Cancer Discov..

[2] Sánchez Y, Huarte M (2013). Long Non-Coding RNAs: Challenges for Diagnosis and Therapies. Nucleic Acid Ther..

[3] Neguembor MV, Jothi M, Gabellini D (2014). Long noncoding RNAs, emerging players in muscle differentiation and disease. Skelet. Muscle.

[4] Guttman, M. & Rinn, J. Modular regulatory principles of large non-coding RNAs. *Nature* 4–11 10.1038/nature10887 (2012). 10.1038/nature10887 PMC4197003 22337053

[5] Rinn JL, Chang HY (2012). Genome regulation by long noncoding RNAs. Annu. Rev. Biochem..

[6] Ward M, McEwan C, Mills JD, Janitz M (2015). Conservation and tissue-specific transcription patterns of long noncoding RNAs. J. Hum. Transcr..

[7] Wapinski O, Chang HY (2011). Long noncoding RNAs and human disease. Trends Cell Biol..

[8] Xu C, Yang M, Tian J, Wang X, Li Z (2011). MALAT-1: A long non-coding RNA and its important 3’end functional motif in colorectal cancer metastasis. Int. J. Oncol..

[9] Gupta RA (2010). Long non-coding RNA HOTAIR reprograms chromatin state to promote cancer metastasis. Nature.

[10] Wan Y, Chang HY (2010). HOTAIR: Flight of noncoding RNAs in cancer metastasis. Cell Cycle.

[11] Pontier DB, Gribnau J (2011). Xist regulation and function eXplored. Hum. Genet..

[12] Hung T (2011). Extensive and coordinated transcription of noncoding RNAs within cell-cycle promoters. Nat. Genet..

[13] Puvvula PK (2014). Long noncoding RNA PANDA and scaffold-attachment-factor SAFA control senescence entry and exit. Nat. Commun..

[14] Bischof, O. & Puvvula, P. K. It Takes Four to Tango: Long Noncoding RNA PANDA, SAF-A, Polycomb Repressive Complexes and NF-Y in Senescence Regulation. *Rna Dis*. 1–5 10.14800/rd.855 (2015).

[15] Han L (2015). Low expression of long noncoding RNA PANDAR predicts a poor prognosis of non-small cell lung cancer and affects cell apoptosis by regulating Bcl-2. Cell Death Dis..

[16] Ma P, Xu T, Huang M, Shu Y (2016). Increased expression of LncRNA PANDAR predicts a poor prognosis in gastric cancer. Biomed. Pharmacother..

[17] Peng W, Fan H (2015). Long non-coding RNA PANDAR correlates with poor prognosis and promotes tumorigenesis in hepatocellular carcinoma. Biomed. Pharmacother..

[18] Zhan Y (2016). Up-regulation of long non-coding RNA PANDAR is associated with poor prognosis and promotes tumorigenesis in bladder cancer. J. Exp. Clin. Cancer Res..

[19] Bielli P, Bordi M, Valentina DB, Sette C (2014). Regulation of BCL-X splicing reveals a role for the polypyrimidine tract binding protein (PTBP1/hnRNP I) in alternative 5′ splice site selection. Nucleic Acids Res..

[20] Schor, I. E. *et al*. Perturbation of Chromatin Structure Globally Affects Localization and Recruitment of Splicing Factors. *PLoS One* **7**, (2012). 10.1371/journal.pone.0048084 PMC3495951 23152763

[21] Hennig S (2015). Prion-like domains in RNA binding proteins are essential for building subnuclear paraspeckles. J. Cell Biol..

[22] Ruskin B, Zamore PD, Green MR (1988). A factor, U2AF, is required for U2 snRNP binding and splicing complex assembly. Cell.

[23] Mackereth CD (2011). Multi-domain conformational selection underlies pre-mRNA splicing regulation by U2AF. Nature.

[24] Agrawal AA (2016). An extended U2AF(65)-RNA-binding domain recognizes the 3’ splice site signal. Nat. Commun..

[25] Singh R, Valcarcel J, Green MR (1995). Distinct binding specificities and functions of higher eukaryotic polypyrimidine tract-binding proteins. Science (80-.)..

[26] Saulière J, Sureau A, Expert-Bezançon A, Marie J (2006). The polypyrimidine tract binding protein (PTB) represses splicing of exon 6B from the beta-tropomyosin pre-mRNA by directly interfering with the binding of the U2AF65 subunit. Mol. Cell. Biol..

[27] Llorian M (2010). Position-dependent alternative splicing activity revealed by global profiling of alternative splicing events regulated by PTB. Nat. Struct. Mol. Biol..

[28] Ramos AD (2015). The long noncoding RNA Pnky regulates neuronal differentiation of embryonic and postnatal neural stem cells. Cell Stem Cell.

[29] Conte MR (2000). Structure of tandem RNA recognition motifs from polypyrimidine tract binding protein reveals novel features of the RRM fold. EMBO J..

[30] Lunde BM, Moore C, Varani G (2007). RNA-binding proteins: modular design for efficient function. Nat. Rev. Mol. Cell Biol..

[31] Ong SE, Mann M (2006). A practical recipe for stable isotope labeling by amino acids in cell culture (SILAC). Nat. Protoc..

[32] Rappsilber J, Mann M, Ishihama Y (2007). Protocol for micro-purification, enrichment, pre-fractionation and storage of peptides for proteomics using StageTips. Nat. Protoc..

[33] Michalski A (2011). Mass Spectrometry-based Proteomics Using Q Exactive, a High-performance Benchtop Quadrupole Orbitrap Mass Spectrometer. Mol. Cell. Proteomics.

[34] Olsen JV (2007). Higher-energy C-trap dissociation for peptide modification analysis. Nat. Methods.

[35] Cox J, Mann M (2008). MaxQuant enables high peptide identification rates, individualized p.p.b.-range mass accuracies and proteome-wide protein quantification. Nat. Biotechnol..

[36] Martins LM (2002). The Serine Protease Omi / HtrA2 Regulates Apoptosis by Binding XIAP through a Reaper-like Motif *. J. Biol. Chem..

[37] Livak KJ, Schmittgen TD (2001). Analysis of relative gene expression data using real-time quantitative PCR and. Methods.

