# Supplementary material for: Identification of novel PANDAR protein interaction partners involved in splicing regulation
Source: Sci Rep. 2018 Feb 12;8:2798. doi: 10.1038/s41598-018-21105-6 (PMC5809411; doi:10.1038/s41598-018-21105-6)
Supplement: Supplementary file 1 — Supplementary Information [file 41598_2018_21105_MOESM1_ESM.pdf]

Identification of novel *PANDAR* protein interaction partners involved in splicing regulation

Pospiech, N.[1], Cibis, H. [1], Dietrich, L. [1], Müller, F.[2] , Bange, T.[2,3] , Hennig, S.*[1,4]

[1]Chemical Genomics Centre of the Max-Planck Society, Otto-Hahn-Str. 11, 44227 Dortmund, Germany

[2]Max-Planck Institute of Molecular Physiology, Otto-Hahn-Str. 11, 44227 Dortmund, Germany

[3]Institute of Medical Psychology, LMU Munich, Goethe-Str. 31, 80336 Munich, Germany

[4]Vrije Universiteit Amsterdam, de Boelelaan 1108, 1081HZ Amsterdam, Netherlands

***Corresponding author:**

Dr. Sven Hennig

de Boelelaan 1108

1081HZ Amsterdam, Netherlands

email: s.hennig@vu.nl

Supplemental Material:

Supplemental Figure S1:

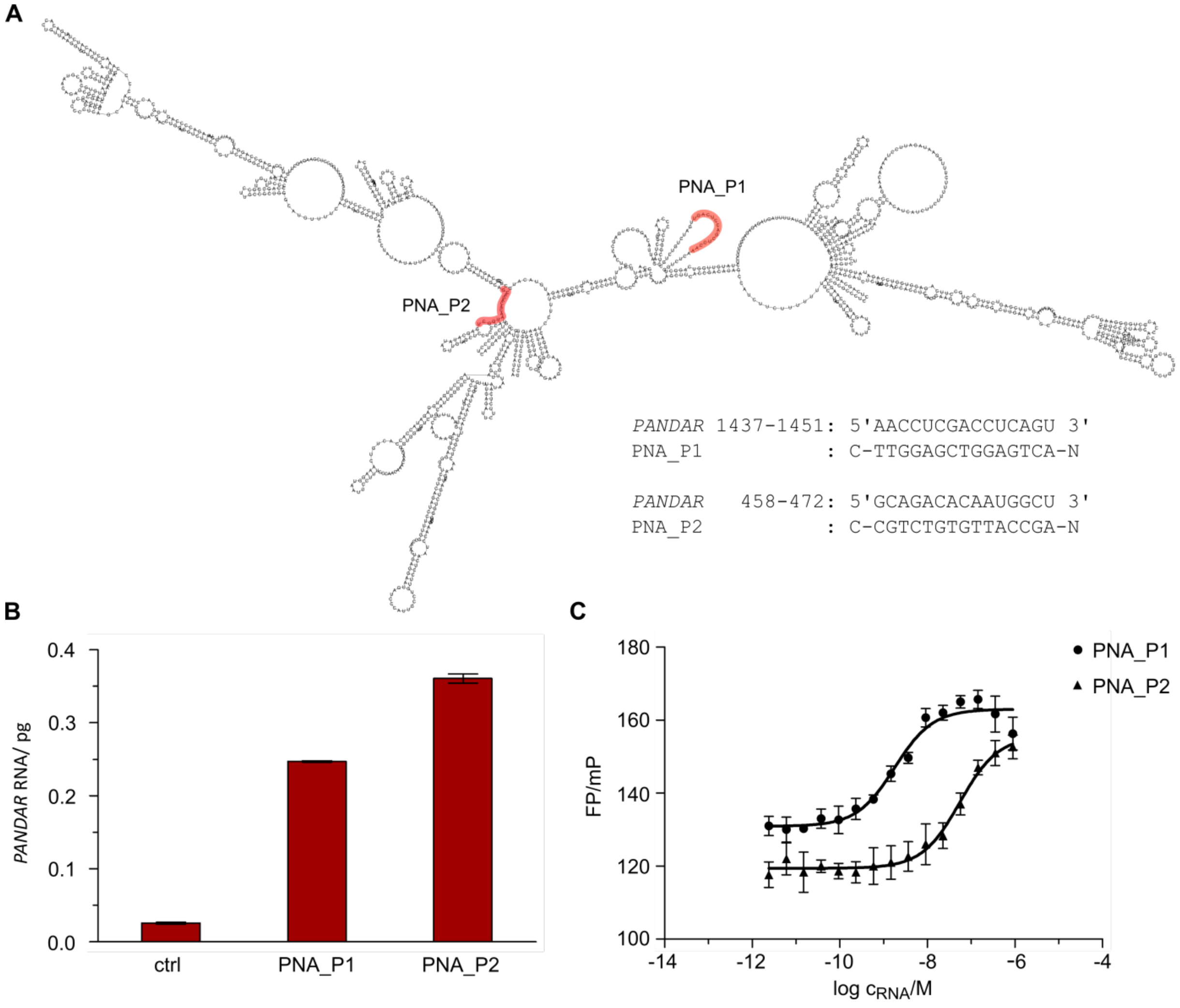

Figure S1: **a**: Secondary structure prediction (mfold) of *PANDAR* RNA. Red lines: binding sites of designed antisense PNA probes. Inlay: sequence of PNA probes with its according *PANDAR* sequence, numbers indicate base pair positions within *PANDAR* full length. **b**: *PANDAR* RNA enrichment from total isolated RNA from human osteosarcoma cells via biotinylated PNA probes. Both PNA probes were able to immobilize *PANDAR* RNA on streptavidin matrix over beads-only control (ctrl). **c**: Analysis of *PANDAR*-PNA binding by fluorescent polarization measurement shows an affinity in the nanomolar range with the best *PANDAR* targeting PNA_P1 (K_D = 1.8 nM).

Supplemental Figure S2:

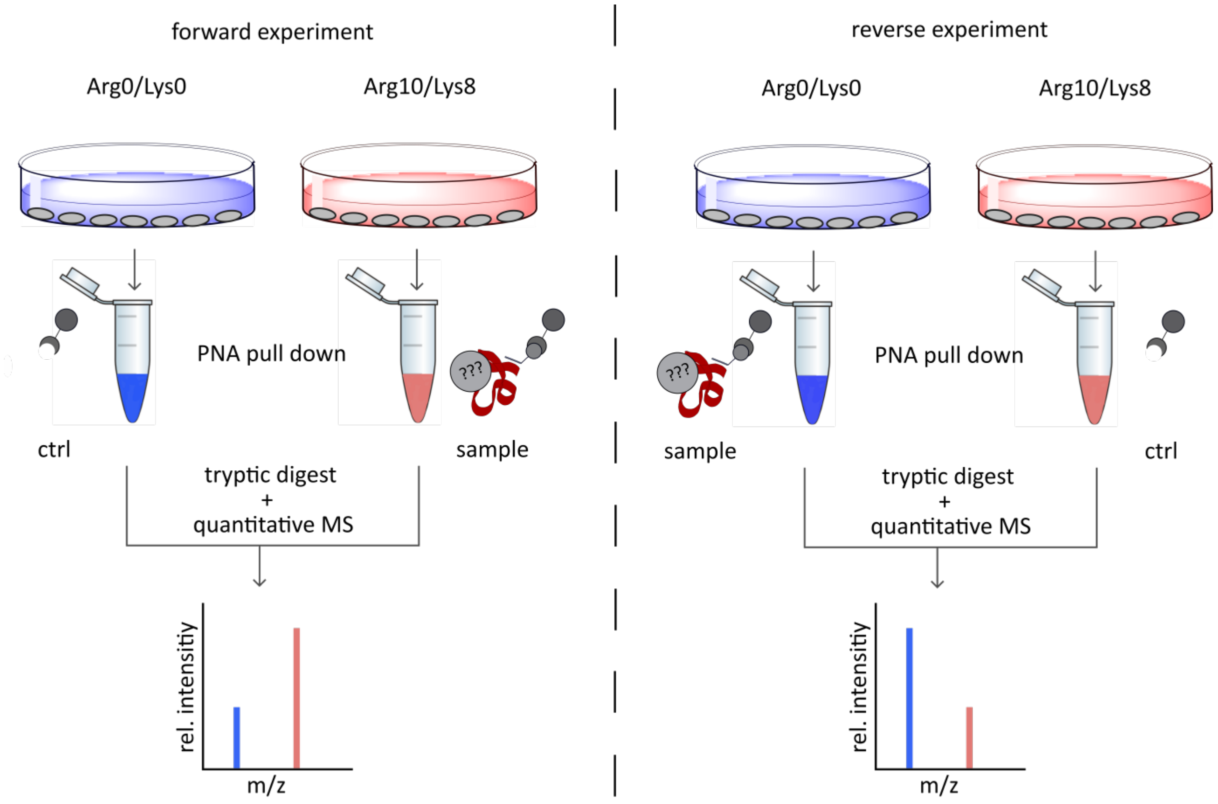

Figure S2: Experimental workflow of PNA-based RNA pull-down combined with SILAC. U2 OS cells were treated with light (indicated by blue color) or heavy SILAC media (indicated by red color) for subsequent quantitative mass spectrometry. Pull-down probe was pre-formed by combining in vitro transcribed *PANDAR* RNA, *PANDAR* targeting biotinylated PNA probe (PNA_P1 or PNA_P2) and streptavidin beads (sample) or beads alone (control). For forward experimental setups (left panel), lysates from U-2 OS cells treated with heavy media were incubated with sample complex and lysate from light cultivated cells was incubated with beads only (control). For reverse experimental setups (right panel), pull-down probes were added vice versa. After tryptic digest of bound proteins quantitative mass spectrometry was performed. Ratios of light and heavy relative peak intensities were calculated for each experiment (= sample/control).

Supplemental Figure S3:

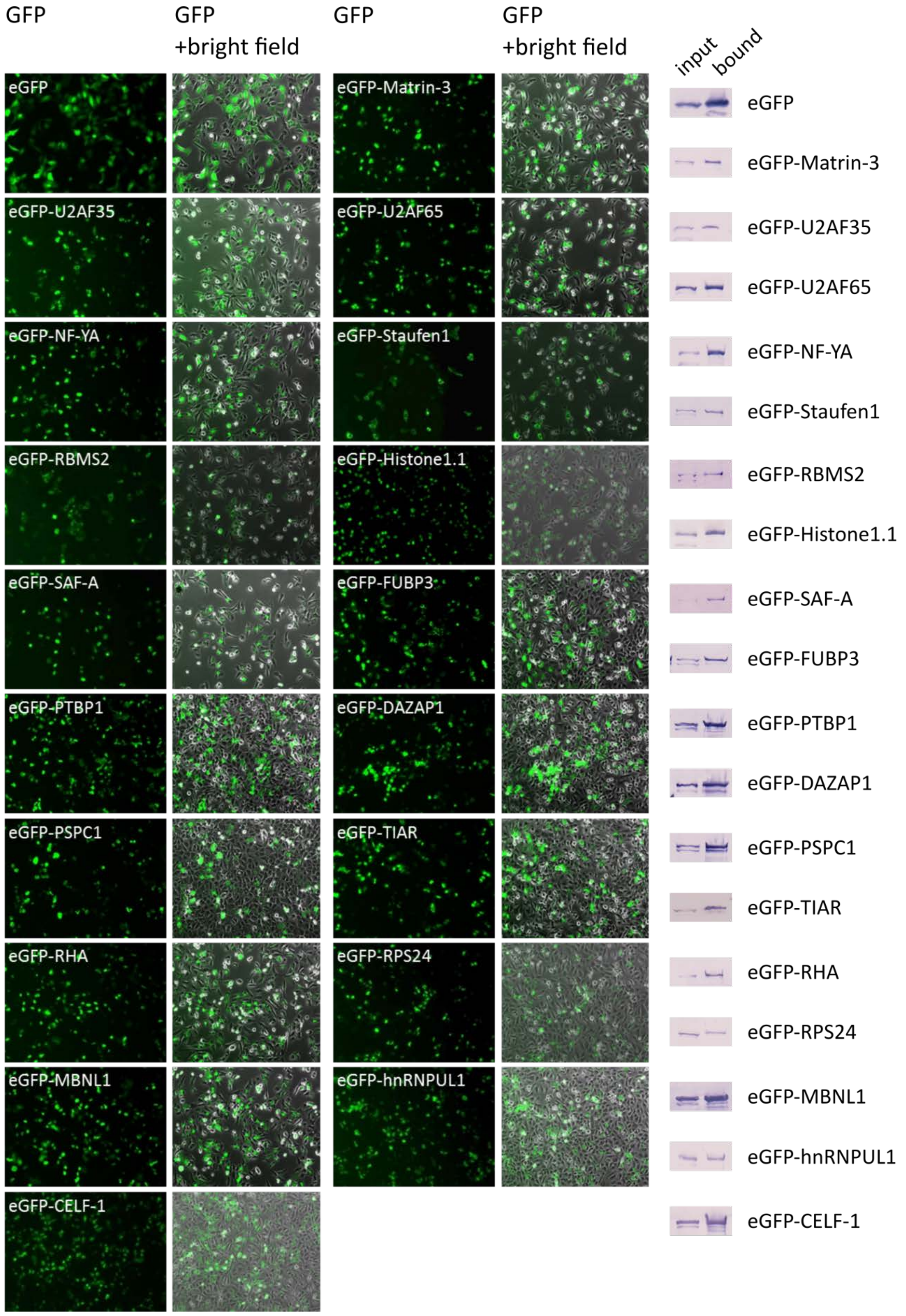

Figure S3: Transfection of *PANDAR* overexpressing U-2 OS cells with hit candidates as eGFP-fusions. Left panel shows eGFP transfected, *PANDAR* overexpressing U2 OS cells including merge of bright field image. Right panel shows western blot analyses of overexpressed and immunoprecipitated full length eGFP-fusion proteins.

Supplemental Figure S4:

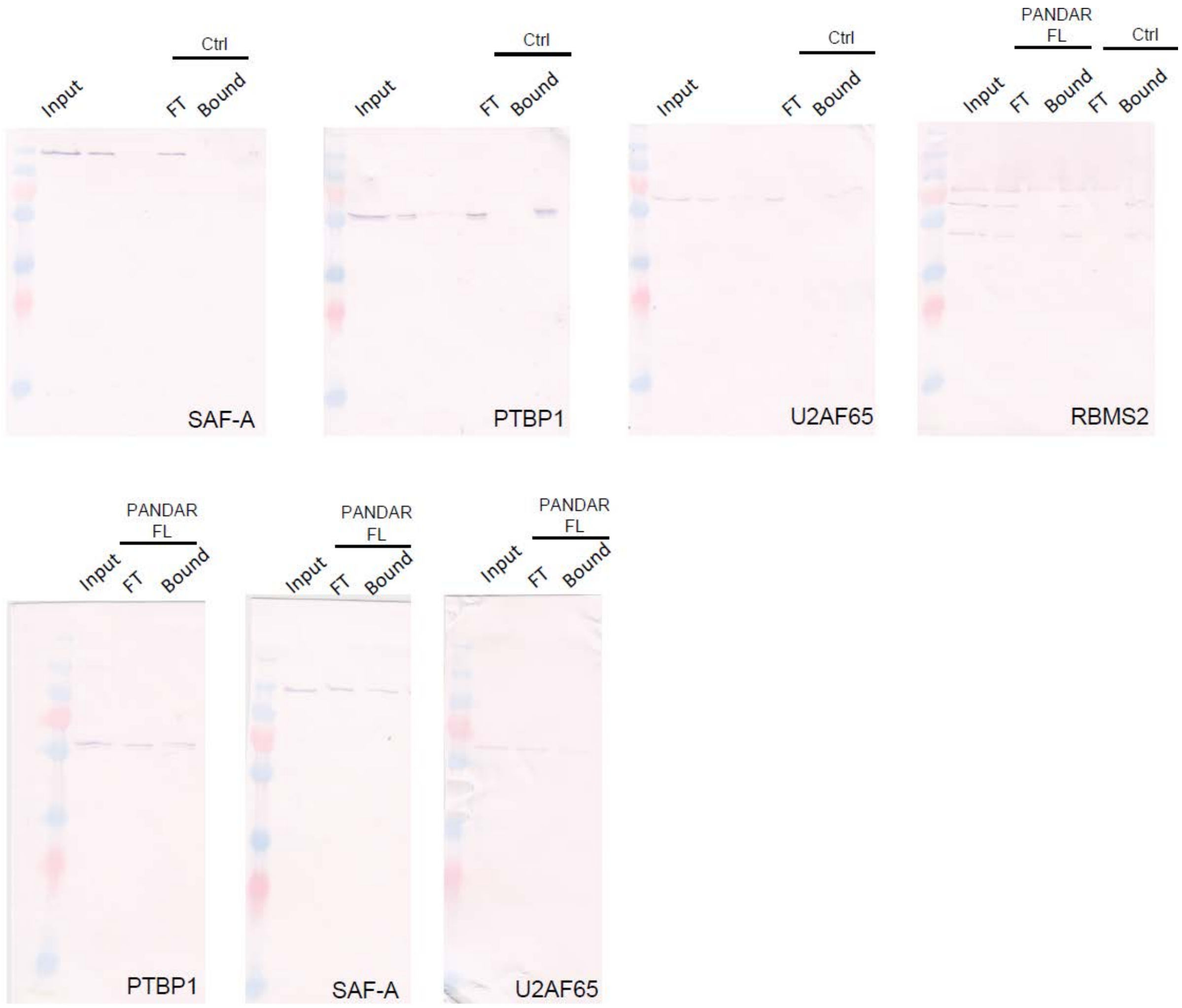

Figure S4: Full scale blots from main figure 2.

Supplemental Figure S5:

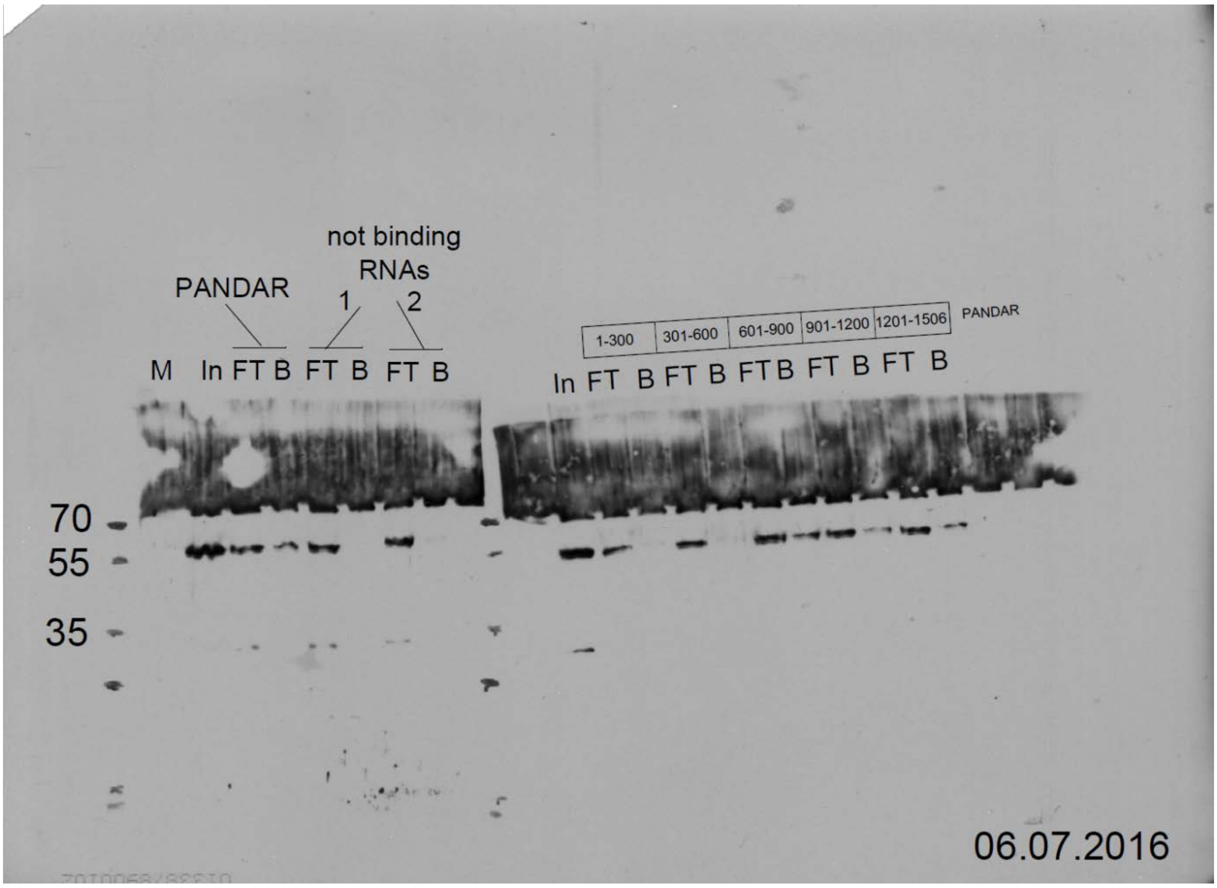

Figure S5: Full scale blots from main figure 3.

Supplemental Figure S6:

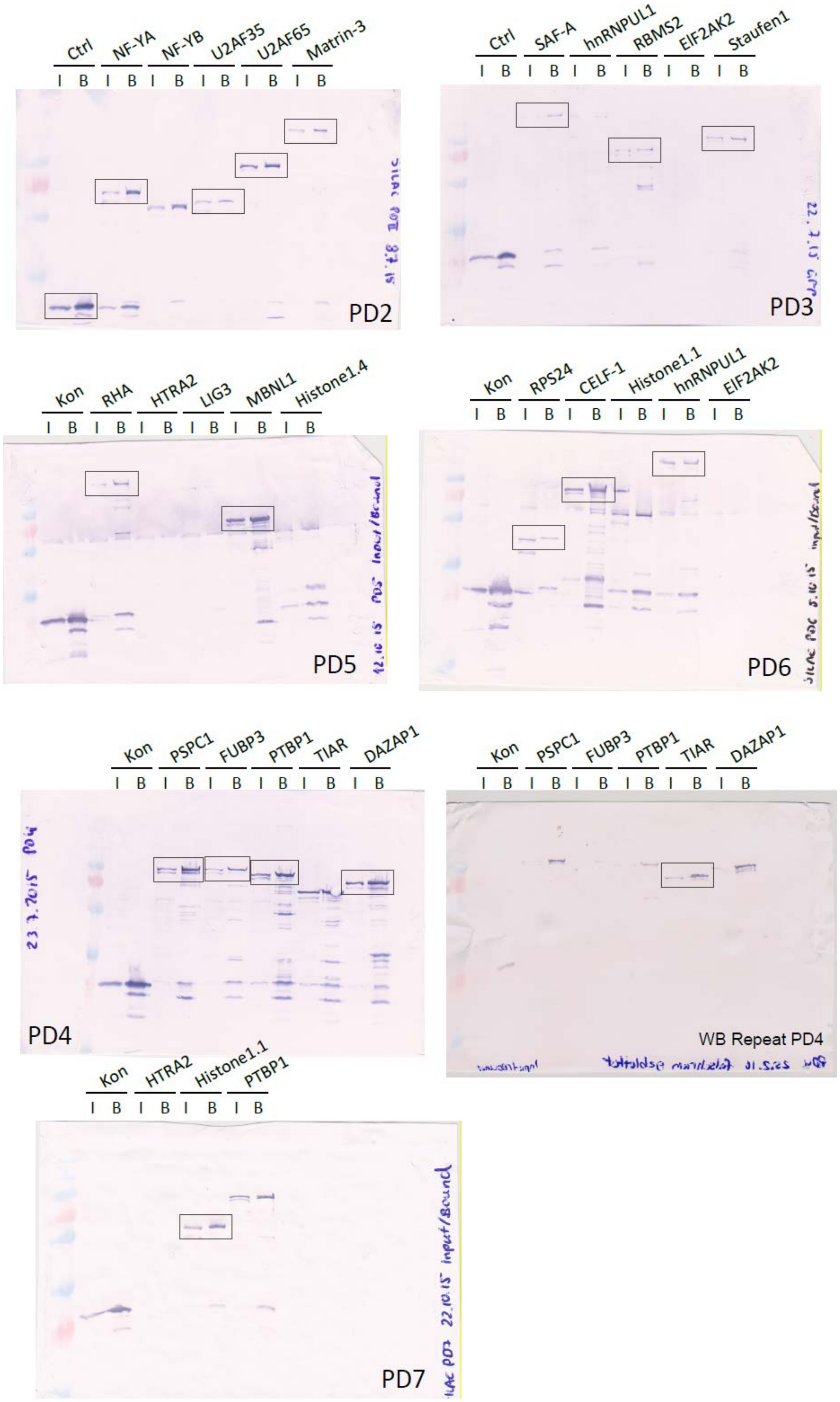

Figure S6: Full scale blots from supplement figure S3.
